# A novel murine model of reversible bile duct obstruction demonstrates rapid improvement of cholestatic liver injury

**DOI:** 10.14814/phy2.14446

**Published:** 2020-05-22

**Authors:** Sarah A. Taylor, Xin‐Yi Yeap, Jiao‐Jing Wang, Kyle D. Gromer, Alyssa Kriegermeier, Richard M. Green, Zheng J. Zhang

**Affiliations:** ^1^ Department of Pediatrics Ann and Robert H Lurie Children’s Hospital of Chicago Chicago IL USA; ^2^ Department of Surgery Northwestern University Chicago IL USA; ^3^ Department of Medicine Northwestern University Chicago IL USA; ^4^ Comprehensive Transplant Center Northwestern University Chicago IL USA

**Keywords:** biliary obstruction, cholestasis, liver injury, liver repair, macrophages

## Abstract

There are limited murine models of cholestatic liver diseases characterized by chronic biliary obstruction and resumption of bile flow. While murine bile duct ligation (BDL) is a well‐established model of obstructive cholestasis, current models of BDL reversal (BDLR) alter biliary anatomy. We aimed to develop a more physiologic model of BDLR to evaluate the time course and mechanism for resolution of hepatic injury after biliary obstruction. In the present study, we restored bile flow into the duodenum without disruption of the gall bladder after murine BDL using biocompatible PE‐50 tubing. After establishing the technique, overall survival for BDLR at 7 or 14 days after BDL was 88%. Sham laparotomy was performed in control mice. Laboratory data, liver histology, and hepatic gene expression were compared among BDL, BDLR, and controls. Laboratory evidence of cholestatic liver injury was observed at day 7 after BDL and rapid improvement occurred within 48 hr of BDLR. After BDLR there was also enhanced gene expression for the bile acid transporter *Abcb11*, however, bile duct proliferation persisted. Assessment of the immune response showed increased gene and protein expression for the general immune cell marker *Cd45* in BDLR versus BDL mice suggesting a reparative immune response after BDLR. In summary, we have established a novel murine model of BDLR that allows for the investigation into bile acid and immune pathways responsible for hepatic repair following obstructive cholestasis. Future studies with our model may identify targets for new therapies to improve outcome in pediatric and adult cholestatic liver disease.

## Graphical abstract

We have established a novel surgical murine model of reversible bile duct ligation that is more physiologic than prior models of bile duct ligation reversal. Our model demonstrates acute hepatic injury from 7‐day bile duct ligation with rapid improvement after restoration of bile flow, including changes in immune, bile acid, and fibrotic pathways during this response. Further studies with this model may identify novel therapeutic targets for pediatric and adult cholestatic liver disease.

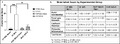

## 1 INTRODUCTION

Obstructive cholangiopathies remain a leading indication for pediatric and adult liver transplantation and represent an unmet need for the development of new therapeutic strategies. There are few effective medical therapies for primary biliary cholangitis (PBC) that slow disease progression and there are no effective medical therapies for primary sclerosing cholangitis (PSC). This is in part due to a lack of pathophysiologic murine models to evaluate the interplay of immune, bile transport, bile metabolism, and fibrotic pathways in response to biliary obstruction and recovery. Murine bile duct ligation (BDL) is an established model of obstructive cholestasis in which there is complete cessation of bile flow. Rupture of the apical membrane of hepatocytes in murine BDL has been shown as a trigger for immune cell infiltration within the first 3 days after murine BDL (Ghallab et al., 2019) and chemokine (C‐C motif) ligand 2 (CCL2)‐mediated cell recruitment contributes to hepatic injury in this model (Cai et al., 2017). In addition, other studies demonstrate impaired chemokine expression in the setting of infection in murine BDL that may contribute to increase susceptibility to infections in cholestasis (Rattay et al., 2018).

Further models to evaluate the time course of obstructive cholestasis and reversal will help address the challenges in maintaining a balanced pro‐ and anti‐inflammatory immune response. Additionally, current murine models of BDL do not replicate the ongoing reparative response that occurs alongside hepatic injury in human diseases in which bile flow is not completely obstructed as can occur in PSC, PBC, and biliary atresia (BA) after Kasai Portoenterostomy. While rat models of partial BDL (Jiang, Li, Wei, Jiang, & Miao, 2016) and reversal after BDL (BDLR) (Huang et al., 2017; Popov et al., 2010) have been established to evaluate the reparative processes in obstructive jaundice, limited models of BDL reversal have been established in mice. To overcome this limitation, murine models of BDL and BDLR have been established, however, prior models have used a cholecystojejunostomy to restore bile flow (Yang et al., 2014). These models alter normal physiology which can affect the dynamics regulating bile acid pool composition similar to has been observed in pediatric patients with progressive familial intrahepatic cholestasis that receive a partial external biliary diversion (Jericho et al., 2015).

In the current study, we establish a novel model of BDLR that recapitulates early changes in histology, bile acid pathways, and the immune response after physiologic restoration of bile flow between the common bile duct and small intestine. This novel model will advance the understanding of specific pathways in hepatic repair after obstructive cholestatic injury and ultimately may help define new target for medical therapies in cholestatic disorders.

## 2 MATERIALS AND METHODS

### 2.1 Murine model of reversible BDL

This study was carried out in strict accordance with the recommendations in the Guide for the Care and Use of Laboratory Animals of the National Institutes of Health. The protocol was approved by the Northwestern University Institutional Animal Care and Use Committee Office. All surgery was performed under combined ketamine and xylazine anesthesia, and all efforts were made to minimize suffering. BDL was performed on C57BL/6 adult (10–12 weeks) male mice using surgical ligation to induce biliary obstruction as previously established (Tag, Sauer‐Lehnen, et al., 2015; Tag, Weiskirchen, et al., 2015). Briefly, a midline incision was made in the abdominal area and the peritoneal cavity was exposed. The intestines were moved toward the lower part of the body with a moistened cotton swab to expose the common bile duct. The bile duct was then separated from the portal vein and hepatic artery. An 8‐0 nylon suture was placed underneath the bile duct and secured with two surgical knots. The abdominal cavity was then closed in two layers.

**FIGURE 1:**
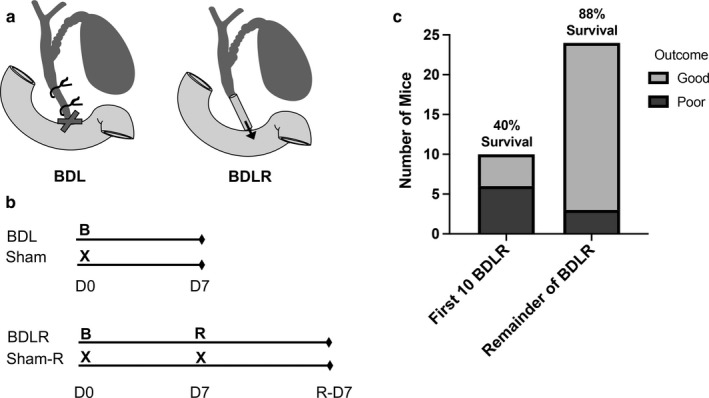
Experimental murine model of reversible bile duct ligation (BDL). (a) BDL surgery was performed by placing two knots on the common bile duct. Reversal was achieved by using a PE‐50 biocompatible tubing to connect the bile duct to the small intestine. (b) Experimental groups included BDL and BDLR mice with sham surgeries performed in parallel with each surgery in the experimental groups (B—bile duct ligation surgery, R—reperfusion surgery, X—sham laparotomy). (c) After establishing the technique of BDLR, overall survival after 7‐day and 14‐day for BDLR surgery reached 88%

BDLR was achieved at 7 and 14 days after BDL using biocompatible Intramedic^TM^ Polyethylene Tubing (PE50 by Becton, Dickinson and Company) to restore bile flow between the bile duct and the duodenum while keeping the gallbladder intact (Figure 1a and b). A small incision was made close to the ligated knots from previous BDL surgery. The PE‐50 tubing was inserted into the enlarged bile duct and secured by a 6‐0 silk suture. The distal end of the tubing was inserted into the duodenum and secured with 10‐0 nylon suture. Finally, the peritoneal cavity was rinsed with sterile saline. Sham surgery was performed on control groups for each of BDL and BDLR experimental groups and included an abdominal incision and closure without manipulation of the biliary system. BDL sham surgery was performed at day 0, BDLR sham mice received a sham surgery both at day 0 and at day 7.

### 2.2 Evaluation of liver injury: biochemical evaluation and immunohistochemistry

Laboratory data were collected at 2, 5, and 7 days after BDL and BDLR including serum alanine aminotransferase (ALT), alkaline phosphatase (ALP), total bilirubin, serum bile acids, and cholesterol values. Liver tissue for histology was obtained from 4 mice at 7 days after surgery for each of BDL, BDLR, and the respective sham groups. Paraffin‐embedded tissue was stained using Hematoxylin and Eosin (H&E), Picro‐Sirius Red, anti‐CD45 (Abcam, ab10558), anticytokeratin 19 (CK19) (Developmental Studies Hybridoma Bank, TROMA‐III), anti‐Ki67 (Abcam, ab16667), anti‐CD68 (Abcam, ab125212), anti‐F4/80 (Cell Signaling, 70076), and anti‐Ly6g (BD Biosciences, 551459). Comparison in staining between groups was made by determining percent area of positive stain (CK19 and Picro‐Sirius Red) and cell counts (Ki67, CD45, CD68, F4/80, and Ly6g). Quantification was performed on five representative 20× fields centered on a portal tract for each of the 4 mice per experimental group for all stains. Mean values per mouse were compared as described in statistical methods. ImageJ Color Deconvolution tool was used to optimize the Ki67 stain. Cell counts were performed with the ImageJ function Analyze Particles and percent area was determined for the CK19 and Sirius Red stains using the Measure function.

To characterize differences in the severity of inflammatory liver injury, two blinded investigators (RG and AK) scored the H&E slide for each of the 4 mice per experimental group by the Ishak scoring system (Goodman, 2007). Each slide was evaluated at 10× magnification and a score was assigned for periportal or periseptal interface hepatitis (0–4), confluent necrosis (0–6), focal lytic necrosis (0–4), and portal inflammation (0–4) (Goodman, 2007). The average of the two reviewer scores for each category were calculated and summed to generate the cumulative Ishak grade (0–18) for statistical comparison between groups.

### 2.3 Characterization of changes in inflammatory, bile acid, and fibrotic pathways by RT‐PCR

We analyzed gene expression for four mice within each experimental and control group at day 7 after BDL, BDLR, or respective sham surgery/surgeries. Quantitative reverse transcription polymerase chain reaction (RT‐PCR) was performed on whole liver RNA as previously described (Liu et al., 2015). Quantitative real‐time PCR (qPCR) was performed with specific primers for genes involved in inflammation (*Cd45, Cd68, Emr1, Ly6g, Ccl2, Tnf‐α, Il1b, Ifng, Il33,* and *Il6*), bile acid metabolism (*Abcb11, Ntcp, Cyp7a1,* and *Mrp2*), and fibrosis (*Acta2, Col1a1,* and *Tgfb1*) (Table S1). Fold change (2^ΔCt^) of our samples was calculated relative to the BDL sham samples and normalized to the housekeeping gene *Gapdh*.

### 2.4 Statistical analysis

Serum liver chemistry values are presented as mean with standard deviation (*SD*). Statistical comparisons between laboratory data were performed using unpaired students *T* test at specific time points. ANOVA with the Bonferroni post hoc test for multiple comparisons was used to evaluate differences between mean gene expression and histology data between groups. Comparison groups included each experimental group and their respective control, the two experimental groups, and the two control groups. Significance is reported for *p* values <.05 (*), *p* < .01 (**), *p* < .001 (***), and *p* < .0001 (****).

## 3 RESULTS

### 3.1 Surgical outcomes

BDLR was performed on a total of 34 mice receiving BDL. After establishing the technique, BDLR was performed with an overall survival of 88% (Figure 1c). More specifically, BDLR at 7 days achieved 93% survival (*n* = 15) and BDLR at 14 days achieved 78% survival (*n* = 9). Poor surgical outcomes occurred in a total of 9 mice, of which 67% (*n* = 6) occurred during the first 10 surgeries. Complications included anesthesia‐related effects, biliary leak, and portal vein tear.

### 3.2 Rapid improvement in biochemical markers of hepatocyte injury after BDLR

**FIGURE 2:**
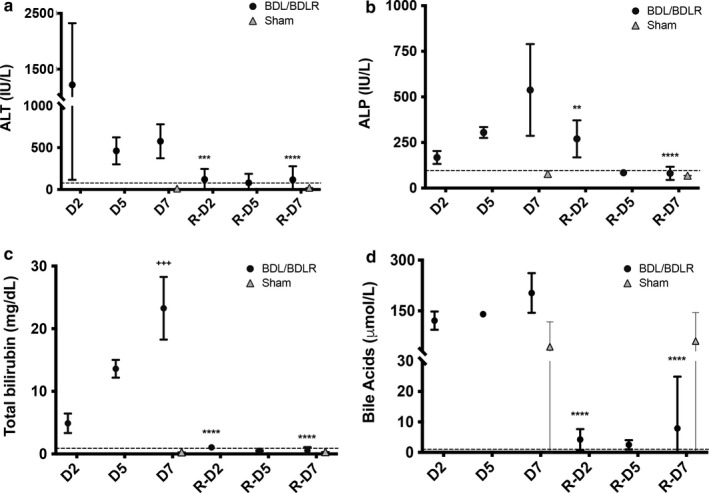
Serum liver chemistries after BDL and BDLR. (a and b) Serum alanine aminotransferase (ALT) and alkaline phosphatase (ALP) values rapidly improved following BDLR in mice. (c and d) Total serum bilirubin and bile acid levels also quickly normalized. Serum bilirubin levels increased between day 2 (D2) and day 7 (D7) with *p* < .001. All serum liver chemistries were significantly improved at reversal day 2 (R‐D2) and 7 (R‐D7) compared to D7. Mean laboratory values after sham surgery were normal for all values except serum bile acids. Upper limit of normal is depicted by a dotted line for each biochemical test (ALT = 77 IU/L, ALP = 96 IU/L, total bilirubin = 0.9 mg/dl, and bile acid < 1 μmol/L). ^+^Indicates comparison to D2; *indicates comparison to D7. **p < .01, ***p < .001, ****p < .0001; ^+++^ *p* < .001

We initially evaluated the timeframe of hepatic recovery using serum laboratory markers for cholestatic liver injury after BDL and BDL reversal. Significant improvement in ALT, ALP, total bilirubin, serum bile acids, and cholesterol occurred at day 2 after reversal as compared to day 7 after BDL, *p* < .01 for all (Figure 2). Comparison of 7‐day laboratory data demonstrated improvement of ALT from 576 ± 203 IU/L (*n* = 5) 7 days after BDL to 116 ± 161 IU/L (*n* = 27) at 7 days after reversal (*p* = 3.9 × 10^–6^) (Figure 2a). Mean ALP also significantly improved over this timeframe from 538 ± 251 IU/L (*n* = 14) to 81 ± 36 IU/L (*n* = 27) at day 7 after reversal (*p* = 1.6 × 10^–11^) (Figure 2b). Total bilirubin was elevated to 23.3 ± 5.0 mg/dl (*n* = 14) at 7 days after BDL and improved to 0.55 ± 0.54 mg/dl (*n* = 27) 7 days after reversal (*p* < 1.0 × 10^–15^) (Figure 2c). Mean bile acid level at day 7 after BDL was 203 ± 59 μmol/L (*n* = 7) which decreased to a mean of 7.9 ± 17 μmol/L (*n* = 26) at 7 days after reversal (*p* < 1.0 × 10^–15^) (Figure 2d). Mean cholesterol levels also significantly improved from 512 ± 164 mg/dl (*n* = 11) at 7 days after BDL to 111 ± 23 mg/dl (*n* = 27) at 7 days after reversal (*p* = 8.0 × 10^–15^, data not shown). Mean biochemical markers of liver injury were all below the upper limit of normal in mice after sham surgery except serum bile acids due to variability among a small sample size (Figure 2). Overall, our data support the ability for significant recovery of bile acid transport after BDL reversal with a reduction in hepatocellular damage.

### 3.3 Hepatic histologic changes after BDL and BDLR

**FIGURE 3:**
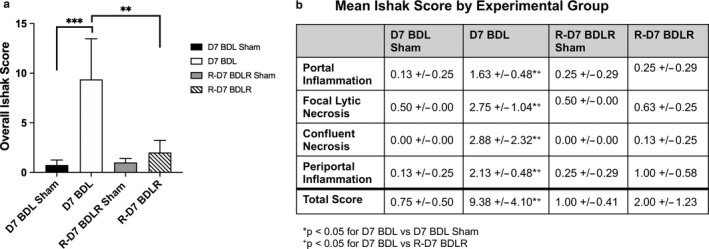
Improvement in acute histologic changes after BDLR. (a) Total Ishak score for H&E slides across all groups demonstrated significantly worse inflammatory liver injury in BDL mice as compared to both their sham controls and BDLR mice. (b) Individual scores for portal inflammation, focal lytic necrosis, confluent necrosis, and periportal inflammation were all significantly worse in BDL mice compared to BDLR. **p < .01, ***p < .001

**FIGURE 4:**
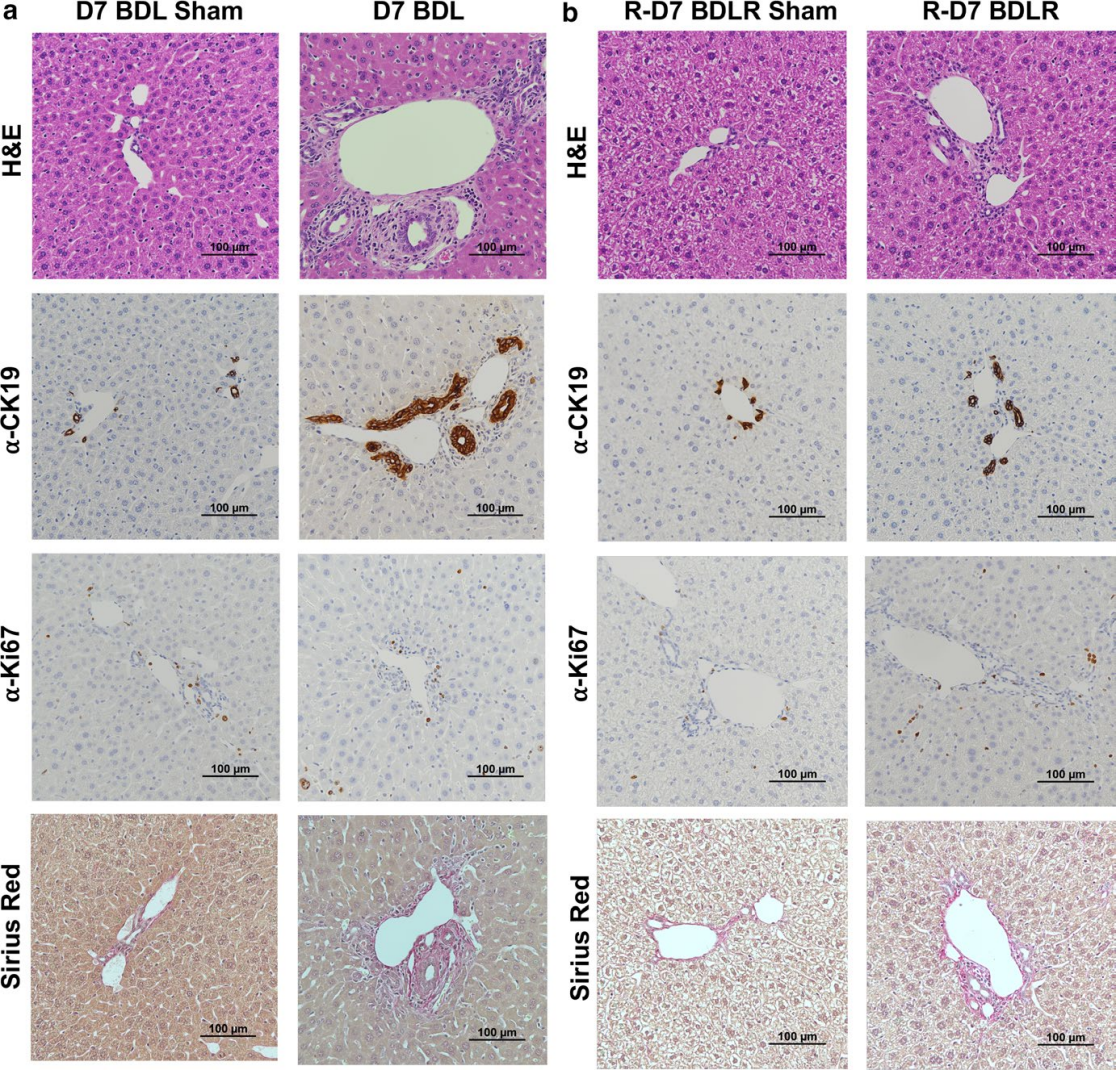
Histologic changes after BDL and BDLR. (a) Hematoxylin & Eosin (H&E) and immunohistochemistry of livers in BDL mice demonstrated hepatocyte injury with a portal infiltrate and increased numbers of CK19‐positive bile ducts. (b) In contrast, BDLR mice demonstrated minimal hepatocyte injury, portal infiltrate, and bile duct proliferation. Overall, Ki67 staining for cell proliferation was sparse across all groups and Sirius red staining showed minimal fibrosis in all mice

**FIGURE 5:**
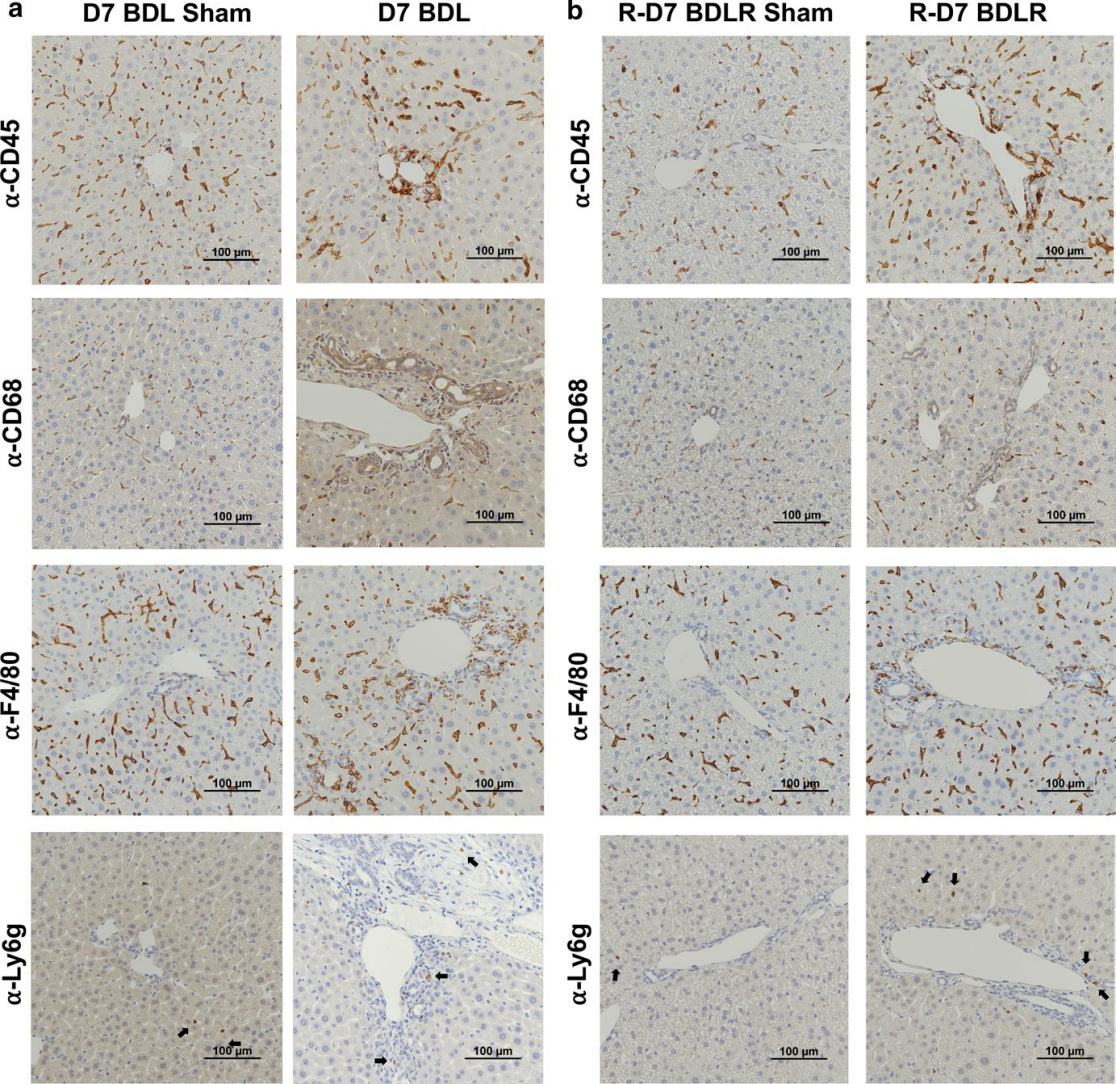
Characterization of immune cell infiltrate by immunohistochemistry. (a) Prominent staining for CD45‐positive immune cells and macrophages (α‐CD68 and α‐F4/80) was observed in the portal tracts in BDL mice rather than the parenchyma as seen in sham controls. (b) The portal infiltration of immune cells appeared reduced in BDLR mice. Ly6g‐positive neutrophils were sparse across all samples, although increased in BDLR mice compared to sham (a and b)

**FIGURE 6:**
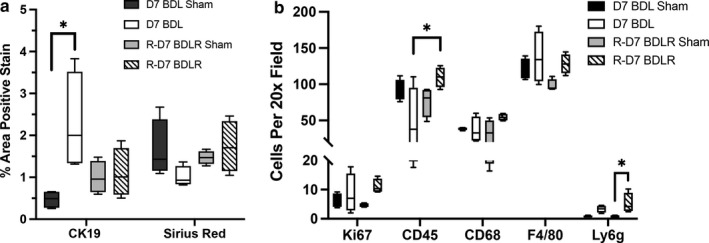
Increased numbers of CD45‐positive cells in BDLR mice. (a) Quantification of the percent area of CK19‐positive stain was significantly increased in BDL mice versus sham controls. There was no difference in percent of positive Sirius Red stain between groups. (b) Cell quantification demonstrated increased numbers of CD45‐positive cells in BDLR mice compared to BDL. Ly6g‐positive cells were increased only in BDLR mice versus their sham controls. *p < .05

Scoring of H&E slides from liver tissue of all experimental groups demonstrated significant inflammatory injury by Ishak scoring criteria after BDL for 7 days (Figure 3). This was significantly improved in BDLR mice to near normal values (Figure 3). Imaging of H&E slides demonstrated hepatocyte injury after BDL with portal expansion, inflammatory cell infiltrate, hepatocyte ballooning, and lobular disarray (Figure 4). As expected before 21–28 days after BDL (Tag, Weiskirchen, et al., 2015), minimal portal fibrosis by Sirius red staining was present across groups without a significant difference in quantification (Figures 4a and 6a). Staining with anti‐CK19 demonstrated an increase in bile duct proliferation in portal tracts of BDL mice versus controls (Figure 4a). Quantification of the percent of CK19‐positive area using ImageJ confirmed significantly increased CK19‐positive staining only in BDL mice at 7 days compared to sham controls (*p* = .0123) (Figure 6a). There was a low number of Ki67‐positive cells without any difference in quantification across groups (Figures 4 and 6b). Interestingly, despite portal damage and increased inflammation on H&E in BDL mice (Figure 4), the number of CD45‐positive cells was only significantly increased in BDLR mice as compared to BDL mice (*p* = .0264) (Figures 5 and 6b). Although low in number overall, increased numbers of Ly6g‐positive cells were present in BDLR versus their sham controls (*p* = .0212) (Figures 5 and 6b). There were no differences in number of CD68 or F4/80‐positive cells between groups (Figures 5 and 6b).

### 3.4 Increased expression of proinflammatory genes persists after BDLR

**FIGURE 7:**
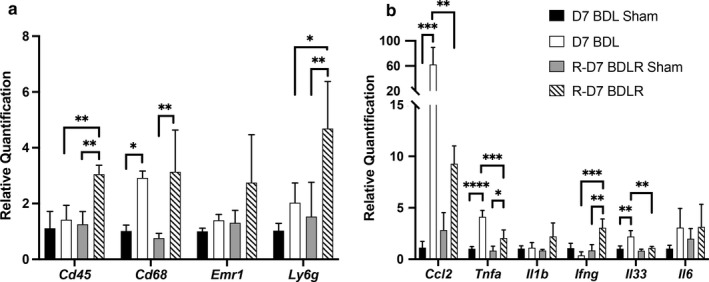
Changes in inflammatory gene expression. (a) CD45 and Ly6g gene expression were significantly increased in BDLR mice compared to BDL and sham controls. However, *Cd68* was increased in both BDL and BDLR mice compared to their controls, respectively, whereas *Emr1* gene expression was not different between groups. (b) The proinflammatory genes *Ccl2, Tnf‐*α, and *Il33* were significantly increased in BDL mice, whereas *Ifn‐*γ was increased in BDLR mice. *p < .05, **p < .01, ***p < .001, ****p < .0001

We next evaluated the gene expression for immune cell markers and inflammatory pathways that may contribute to the histologic changes between groups. Notably, qPCR of liver homogenate demonstrated that gene expression for *Cd45* encoding the leukocyte common antigen was significantly increased in BDLR mice versus both BDL mice and BDLR sham controls (Figure 7a). *Ly6g* gene expression was also increased in BDLR mice (Figure 7a). *Emr1* (F4/80) showed no difference between groups, whereas *Cd68* gene expression was significantly increased in both BDL and BDLR mice compared to their sham controls in contrast to protein expression on histology (Figure 7). Gene expression for *Ccl2, Tnfa,* and *Il33* was significantly increased in BD mice compared to their sham controls, whereas there was no difference in *Il6* between groups (Figure 7b). While levels for *Ccl2, Tnfa,* and *Il33* were significantly decreased in BDLR mice, *Ccl2* and *Tnfa* remained higher than their controls (Figure 7b). In contrast, gene expression for *Ifng* was increased in BDLR versus BDL mice suggesting an alternate inflammatory pathway than the other cytokines (Figure 7b).

### 3.5 Hepatocyte adaptation in bile acid metabolism and fibrotic pathways

**FIGURE 8:**
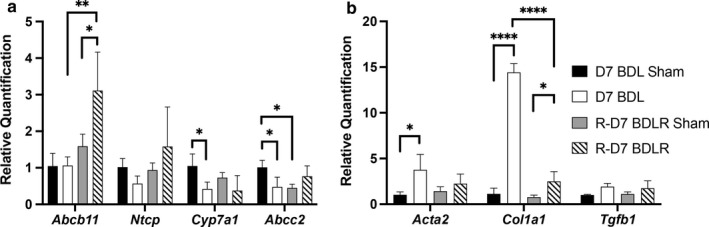
Gene expression of bile acid metabolism genes and mediators of fibrosis. (a) BDL mice had significantly decreased gene expression for *Cyp7a1* and *Abcc2* compared to their sham controls. In contrast, *Abcb11* was significantly increased after BDLR versus BDL. (b) Increased gene expression for Collagen 1 (*Col1a1*) was present in BDL mice compared to both their sham controls and BDLR mice. Increased expression of *Acta2* was observed only in BDL mice. *p < .05, **p < .01, ***p < .001, ****p < .0001

We characterized regulation of specific hepatic transporters to define the pathways that improve after BDLR, potentially reducing the toxic effect from bile acid accumulation. Relative quantification of bile acid transporter gene expression showed a significant decrease in gene expression for the bile acid synthesis gene *Cyp7a1* and the liver transporter Mrp2 (*Abcc2)* in BDL mice compared to their sham controls, however, expression of *Abcc2* was also significantly different between sham controls (Figure 8a). None of these changes persisted in BDLR mice (Figure 8a). Interestingly, gene expression of the bile acid export pump *Abcb11* was significantly increased in BDLR mice compared to both their sham controls and BDL mice (Figure 8a).

Lastly, while we did not find increased fibrosis histologically at 7 days after BDL in line with prior reports (Tag, Weiskirchen, et al., 2015), we evaluated the expression of genes involved in fibrosis that encode alpha‐smooth muscle actin (*Acta2*), collagen 1 (*Col1a1*), and TGF‐beta (*Tgfb1*). *Acta2* was significantly increased in BDL mice as compared to sham controls, whereas *Tgfb1* showed no difference between groups (Figure 8b). In contrast, while *Col1a1* remained significantly elevated in both BDL and BDLR mice versus their sham controls, BDLR mice had significantly reduced expression for *Col1a1* as compared to BDL mice (Figure 8b). This finding suggests that upregulation of profibrotic genes occurs early before histologic changes and that there may be rapid reduction in expression of these genes after alleviation of biliary obstruction. Further studies using our model of reversal at longer time intervals from BDL surgery will more fully evaluate the changes in fibrotic pathways.

## 4 DISCUSSION

We have developed a novel murine model that represents more physiologic restoration of bile flow after obstruction than prior models using a cholecystojejunostomy (Yang et al., 2014). Prior work using the cholecystojejunostomy murine model of BDL reversal explored mechanisms of fibrosis after BDL for 14 days (Yang et al., 2014). Our murine model of the acute phase of hepatic injury after BDL for 7 days shows a rapid improvement in cholestatic inflammatory liver injury after restoration of bile flow that has not previously been demonstrated. Scoring by Ishak criteria strongly supports the efficacy of our model to induce the severe inflammatory damage known to occur within 1 week after BDL (Tag, Weiskirchen, et al., 2015). Notably, serum aminotransferases, total bilirubin, and bile acids all significantly improved by 2 days after BDLR thereby demonstrating the capacity for rapid improvement of hepatic immune and bile transport pathways after alleviation of biliary obstruction. As fibrosis after BDL takes 3–4 weeks to develop (Tag, Weiskirchen, et al., 2015), we demonstrate no significant increase in fibrosis by histology in BDL mice at 7 days. However, our gene expression data support that stimulation of specific profibrotic pathways may occur prior to histologic findings. Further studies using our model at additional time points will better define the interrelated molecular pathways in response to biliary obstruction and may identify therapeutic targets to promote hepatic recovery.

While prior work has described the inflammatory changes in BDL, we provide important insight into the immune response after restoration of bile flow that may have significant application to human disease. Recent studies in murine BDL have shown that the influx of CD45+ immune cells near the site of bile infarcts occurs early, along the same timeframe as liver enzyme elevation, hepatocyte death, and release of damage‐associated molecular patterns (Ghallab et al., 2019). While the present study does not evaluate the microscopic changes in immune cell infiltration, our global assessment by histology and gene expression provides insight into the importance of similarly tracking temporal changes after restoration of bile flow. Interestingly, despite recruitment of immune cells at the level of the canaliculi after BDL (Ghallab et al., 2019), we demonstrate increased protein and gene expression for CD45 in BDLR mice as compared to BDL. In contrast, gene expression for the proinflammatory cytokines *Ccl2, Tnfa,* and *Il33* was significantly reduced in BDLR versus BDL mice thereby suggesting a prorestorative role for CD45‐positive immune cell subsets after resolution of biliary obstruction. Interestingly, gene expression for interferon‐gamma (IFN‐γ) was increased in BDLR mice compared to their sham controls and BDL mice, thereby suggesting a more immune regulatory role for IFN‐γ in hepatic recovery than the commonly accepted proinflammatory role (Zhang, 2007). While there were no significant differences in either macrophage marker CD68 or F4/80, macrophages recruited by Ccl2 are highly heterogeneous and have subset‐specific roles in response to specific environmental cues that may not be identified in the current study (Brempelis & Crispe, 2016; Ju & Tacke, 2016; Lavin et al., 2014; Scott et al., 2016). Further work is thus needed to more precisely define the immune cell subsets responsible for hepatic injury and repair over the time course of BDL and BDL reversal.

Similar to previous studies demonstrating adaptation of bile acid transporters after BDL (Donner & Keppler, 2001; Gartung et al., 1996; Trauner et al., 1997; Zhang et al., 2012), we show that rapid regulation of bile acid transporters also occurs in a short timeframe after restoration of bile flow. Decreased gene expression for Cyp7a1, the rate‐limiting protein in bile acid synthesis after BDL is consistent with the known compensatory mechanism to attempt to decrease the bile acid pool and hepatic bile acid concentration in the setting of biliary obstruction regulated by Farnesoid X receptor (FXR) bile acid signaling. While there is no change in *Abcb11* gene expression encoding for the bile salt export pump (BSEP) after BDL, *Abcb11* levels significantly increased in BDLR mice suggesting hepatic BSEP may be an important adaptive bile acid transporter to restore bile flow. This finding contrasts with previous studies in which BSEP‐deficient mice do not develop significant cholestatic liver injury in the setting of bile duct ligation (Fuchs et al., 2017) suggesting BSEP is not central to maintaining a nontoxic murine bile acid pool in this genetically modified mouse model. The phenotype of murine BSEP deficiency markedly differs from human BSEP deficiency since children lacking hepatic BSEP have Progressive Familial Intrahepatic Cholestasis Type 2 (PFIC2) which can lead to cirrhosis and liver failure at a young age. Our BDLR model provides an opportunity for translational studies examining the significance of the adaptive role for BSEP after restoration of bile flow.

While our study develops a novel model of biliary obstruction to investigate the hepatic and immune response after restoration of bile flow, our findings need to be expanded to include additional time intervals to more fully define mechanistic changes in bile duct ligation followed by reversal. In line with prior studies demonstrating time intervals of 3–4 weeks after BDL for evaluation of fibrosis (Tag, Weiskirchen, et al., 2015), there was a lack of significant fibrosis on Sirius red staining after BDL for 7 days. Despite this, we found significant changes in fibrogenic gene expression at 7 days both before and after reversal, thereby demonstrating the important role for this model may serve at later time points to increase our understanding of hepatic fibrosis. Lastly, future studies isolating specific immune cell populations will identify the cell subsets responsible for the inflammatory response.

We demonstrate a novel murine model of bile duct ligation followed by reversal of obstruction which demonstrated evidence of rapid improvement in hepatocyte injury and inflammation. However, despite normalization of serum liver chemistries of cholestasis and hepatic injury, histologic evidence of biliary obstruction persists for at least 7 days. This supports the need for such models of the reparative hepatic response to identify targets that may hasten hepatic recovery. Further research using this model will further define the specific cells regulating changes in the bile acid, immune, and fibrotic pathways to ultimately identify new cell‐specific therapeutic targets for pediatric and adult cholestatic liver disease.

## CONFLICT OF INTEREST

The authors report no conflicts of interest.

## AUTHOR CONTRIBUTIONS

Study concept and design (SAT, RMG, ZJZ); acquisition of data (SAT, XY, JW, KDG, AK, RMG, ZJZ); analysis and interpretation of data (SAT, XY, JW, KDG, AK, RMG, ZJZ); drafting of the manuscript (SAT); critical revision of the manuscript for important intellectual content (SAT, XYY, JJW, KDG, AK, RMG, ZJZ).
