# Supplementary material for: A novel murine model of reversible bile duct obstruction demonstrates rapid improvement of cholestatic liver injury
Source: Physiol Rep. 2020 May 22;8(10):e14446. doi: 10.14814/phy2.14446 (PMC7243199; doi:10.14814/phy2.14446)
Supplement: Supplementary file 1 [file PHY2-8-e14446-s001.docx]

**Supplemental Table.** Primer sequences for RT-PCR.

| **Gene** | **Forward Sequence** | **Reverse Sequence** |
| --- | --- | --- |
| *Gapdh* | AGGTCGGTGTGAACGGATTTG | TGTAGACCATGTAGTTGAGGTCA |
| *Cd45* | ATGGTCCTCTGAATAAAGCCCA | TCAGCACTATTGGTAGGCTCC |
| *Cd68* | CGCAGACGACAATCAACCTA | AGTGGCATGGTGAAGAGATG |
| *Emr1* | TGACTCACCTTGTGGTCCTAA | CTTCCCAGAATCCAGTCTTTCC |
| *Ly6g* | CTTCTCTGATGGATTTTGCGTTG | AGTAGTGGGGCAGATGGGAAG |
| *Ccl2* | TTAAAAACCTGGATCGGAACCAA | GCATTAGCTTCAGATTTACGGGT |
| *Tnfa* | CCCTCACACTCAGATCATCTTCT | GCTACGACGTGGGCTACAG |
| *Il1b* | GCAACTGTTCCTGAACTCAACT | ATCTTTTGGGGTCCGTCAACT |
| *Ifng* | ATGAACGCTACACACTGCATC | CCATCCTTTTGCCAGTTCCTC |
| *Abcb11* | TCTGACTCAGTGATTCTTCGCA | CCCATAAACATCAGCCAGTTGT |
| *Ntcp* | CAAACCTCAGAAGGACCAAACA | GTAGGAGGATTATTCCCGTTGTG |
| *Cyp7a1* | AGCAACTAAACAACCTGCCAGTACTA | GTCCGGATATTCAAGGATGCA |
| *Abcc2* | GCTTCCCATGGTGATCTCTT | ATCATCGCTTCCCAGGTACT |
| *Acta2* | TCCTCCCTGGAGAAGAGCTAC | TATAGGTGGTTTCGTGGATGC |
| *Col1a1* | AGAGGCGAAGGCAACAGTCG | GCAGGGCCAATGTCTAGTCC |
| *Tgfb1* | ATTCCTGGCGTTACCTTG | CTGTATTCCGTCTCCTTGGTT |
| *Il33* | TGAGACTCCGTTCTGGCCTC | CTCTTCATGCTTGGTACCCGAT |
| *Il6* | CAAAGCCAGAGTCCTTCAGAG | GTCCTTAGCCACTCCTTCTG |
